# Safety of and Cellular Response to Segmental Bronchoprovocation in Allergic Asthma

**DOI:** 10.1371/journal.pone.0051963

**Published:** 2013-01-16

**Authors:** Loren C. Denlinger, Elizabeth A. B. Kelly, Ann M. Dodge, John G. McCartney, Keith C. Meyer, Richard D. Cornwell, Mary Jo Jackson, Michael D. Evans, Nizar N. Jarjour

**Affiliations:** 1 Allergy, Pulmonary and Critical Care Division, Department of Medicine, University of Wisconsin, Madison, Wisconsin, United States of America; 2 Department of Biostatistics and Medical Informatics, University of Wisconsin, Madison, Wisconsin, United States of America; University Hospital Freiburg, Germany

## Abstract

**Rationale:**

Despite its incorporation into research studies, the safety aspects of segmental allergen bronchoprovocation and differences in cellular response among different allergens have received limited consideration.

**Methods:**

We performed 87 segmental challenges in 77 allergic asthma subjects. Allergen dose was based on each subject’s response to whole lung allergen challenge. Bronchoalveolar lavage was performed at 0 and 48 hours. Safety indicators included spirometry, oxygen saturation, heart rate, and symptoms.

**Results:**

Among subjects challenged with ragweed, cat dander, or house dust mite, there were no differences in safety indicators. Subjects demonstrated a modest oxygen desaturation and tachycardia during the procedure that returned to normal prior to discharge. We observed a modest reduction in forced vital capacity and forced expiratory volume in one second following bronchoscopy. The most common symptoms following the procedure were cough, sore throat and fatigue. Total bronchoalveolar lavage fluid cell numbers increased from 13±4 to 106±108×10^4^ per milliliter and eosinophils increased from 1±2 to 44±20 percent, with no significant differences among the three allergens.

**Conclusions:**

In mild allergic asthma, segmental allergen bronchoprovocation, using individualized doses of aeroallergens, was safe and yielded similar cellular responses.

## Introduction

Since the first guidelines addressing the use of bronchoscopy and bronchoalveolar lavage in asthma were published in 1985 [1], segmental bronchoprovocation with allergen (SBP-AG) has been used to investigate mechanisms of allergic airway inflammation [2]. Compared to whole-lung allergen inhalation challenge, depositing allergen directly into a specific lung segment improves the precision of allergen dosing, limits the total area of exposure, and should improve the safety of allergen challenge. SBP-AG also produces a more intense inflammatory response [3], allowing purification and characterization of airway cells. The benefits of this research tool must be weighed against the potential risks of performing an invasive pulmonary procedure in subjects with reactive airway disease. An NIH workshop on bronchoprovocation and investigative bronchoscopy endorsed the continued use of this technique; however, the need for additional safety data was emphasized [4], particularly in light of the death of a research subject that occurred in 1996 after investigative bronchoscopy [5].

The safety aspects of SBP-AG in subjects with atopic asthma were investigated by Krug and colleagues in 1996, using a dust mite or grass allergen dose titration method based on results from skin testing [6]. They reported a 29% incidence of diffuse wheezing among their 49 subjects, 7 of whom required premature termination of the procedure due to respiratory distress. Additionally, 9 subjects had their lowest oxygen saturation during the procedure recorded between 75–85%. In 2001, Moore and colleagues compared the response to SBP-AG between subjects with mild (n = 8) and moderate (n = 10) asthma with a fixed dose of ragweed extract [7]. They noted a modest decline in forced expiratory volume in one second (FEV_1_) and forced vital capacity (FVC) immediately following the bronchoscopy compared to pre-bronchoscopy baseline. The spirometry returned toward baseline 24 hours later and only minimal reduction in spirometry was seen after the second bronchoscopy (without SBP-AG). Julius and colleagues [8] in a study of 78 subjects, found a lower incidence of wheezing after SBP-AG when subjects received an individualized allergen dose compared to those who received a fixed dose. Three subjects required intravenous saline for hypotension and/or dehydration. No increase in adverse events was noted with repeated procedures.

None of these papers included detailed information about symptoms, spirometry, or changes in oximetry or heart rate during and following the procedure. In the study by Julius et al., peak expiratory flow rate (PEFR) was monitored hourly post-SBP-AG in 7 subjects and reported to decrease following SBP-AG compared to baseline; however, spirometry was not included in that study [8]. Finally, although it has been suggested that allergens containing protease activity, such as animal dander and house dust mite, are more likely to induce airway inflammation [9], there are few data available regarding differences in elicited inflammatory cell profiles or safety aspects among different allergens. Thus, we prospectively compared the safety profiles and cellular responses to SBP-AG among three aeroallergens (ragweed (RW), cat dander (CAT), or house dust mite (HDM)) in mild asthma subjects. We believe that further understanding of the safety aspects of SBP will have important implications regarding optimizing safe outcomes and limiting adverse events in future research protocols involving SBP.

## Methods

### Subjects

The studies were approved by the Institutional Review Board of the Human Subjects Committee of the University of Wisconsin. Written informed consent was obtained for all subjects prior to their participation. All allergic asthma subjects who underwent bronchoscopy and SBP-AG at the University of Wisconsin between July 1999 and August 2008 were included in this analysis. Additional details are included in the Supporting Information section (Text S1).

### Allergen Dose Determination

Short ragweed (*Ambrosia artemisiifolia*), house dust mite (*Dermatophagoides farinae*) and standardized cat hair (*Felis catus (domesticus)*) extracts were obtained from Greer Laboratories (Lenoir, NC). Although allergen preparations have previously been associated with endotoxin contamination [10], we had tested similar allergen preparations by the Limulus amoebocyte assay method and found them to be below the level of detection. At least four weeks before bronchoscopy, a graded whole-lung allergen inhalation challenge was performed as described [11], [12] to determine the provocative dose of allergen producing a 20% decrease in FEV_1_ (AG-PD_20_). Baseline spirometry was performed and repeated 10 minutes after five breaths of saline diluent. If FEV_1_ remained within 10% of baseline, five breaths of allergen were inhaled and spirometry was repeated 10 minutes later. Consecutively greater concentrations of allergen were given until FEV_1_ fell by ≥20% from post-diluent and was sustained for at least 20 minutes. Subjects were monitored until FEV_1_ returned to within 10% of baseline. The AG-PD_20_ was calculated by linear interpolation of the last two doses on the dose-response curve. If a 20% drop in FEV_1_ was not achieved after the subject had received the highest allergen concentration, the cumulative dose reached at that point was used as the subject’s AG-PD_20_. Subjects who did not tolerate the whole lung antigen challenge did not undergo SBP.

### Bronchoscopy, BAL and SBP-AG

All subjects were given inhaled albuterol (180 mcg) prior to bronchoscopy. Most subjects received an anxiolytic (intramuscular midazolam 0.5–2.0 mg) and an anticholinergic (intramuscular glycopyrrolate 0.2 mg or atropine 0.5 mg). Nasopharyngeal anesthesia was achieved by topical lidocaine (1% solution, lidocaine gel, lidocaine spray). Additional lidocaine, 1% solution, was administered via bronchoscope, as needed, to control cough. On day 0 (D0), bronchoscopy with BAL was performed in one (n = 13) or two different segments (n = 74) according to the specific study protocol they were enrolled in. After BAL was completed, SBP-AG was performed. In the first segment, approximately 10% of the AG-PD_20_, diluted in 10 ml normal saline, was instilled through the bronchoscope held in wedge position. If this dose was well tolerated, SBP-AG was performed in a second segment using approximately 20% of the AG-PD_20_. After 48 hours (D2), subjects returned for a repeat bronchoscopy with BAL of the same segment(s). Patient monitoring and the methods of BAL processing are described in the Supporting Information section (Text S1).

### Statistical Analysis

Data are presented as medians with first and third quartiles or as rates. Heart rate, SpO_2_, BAL cell counts, and FEV_1_ values were compared among time points and among antigen types (RW, HDM, CAT) using mixed-effects linear models with fixed effects for time and antigen and a random effect for subject to account for the correlation of repeated measures within subjects. Absolute cell counts were log-transformed for analysis. Symptom frequencies were compared among antigen groups using Fisher’s exact test for count data. A *p* value of <0.05 was considered statistically significant. Analyses were performed using R version 2.9.1 (R Foundation for Statistical Computing, Vienna, Austria).

## Results

The median PD_20_ values and interquartile ranges for all three allergens used for whole lung allergen challenge are shown in Table 1. A total of 87 SBP-AG procedures were performed on 77 allergic asthma subjects. Six subjects were challenged on two occasions and two were challenged on three occasions. There were no statistically significant differences in age, sex, methacholine PC_20_, or spirometry among subjects receiving RW (n = 23), HDM (n = 28), or CAT (n = 26) allergens (Table 1). Three subjects did not undergo bronchoscopy on D2 due to development of cold symptoms (one HDM, one RW) or hyperventilation during baseline procedure (one HDM).

**Table 1:** Baseline subject characteristics.

	RW (n = 23)	HDM (n = 28)	CAT (n = 26)
Age (years, median [quartiles])	22 [19,24]	22 [20,26]	23 [20,26]
Gender (# male/# female)	11/12	10/18	16/10
FEV_1_ (% predicted, median [quartiles])	94 [88,100]	94 [87,102]	93 [87,100]
FVC (% predicted, median [quartiles])	103 [99,107]	100 [94,107]	98 [92,104]
FEV_1_/FVC (median [quartiles])	0.80 [0.73,0.85]	0.78 [0.75,0.87]	0.80 [0.76,0.83]
Methacholine PC_20_ (mg/mL, median [quartiles])	2.5 [1.4,10.6]	2.7 [1.2,4.4]	2.9 [0.7,5.4]
Antigen PD_20_ ((B)AU, median [quartiles])	55 [24,123]	31 [13,63]	46 [6,81]

### Heart Rate and SpO_2_

**Figure 1:**
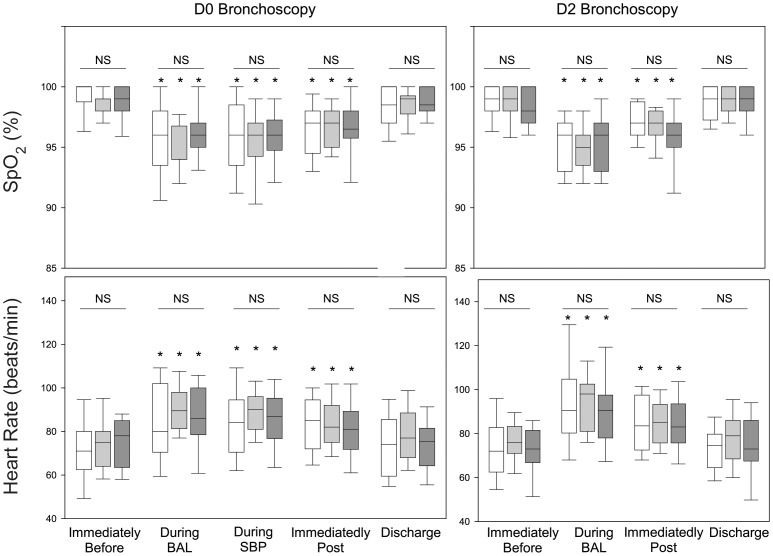
Comparison of oxygen saturation and heart rate on D0 and 48 hours after SBP-AG (D2). On D0, oxygen saturation and heart rate were monitored immediately before initiation of bronchoscopy, during BAL, during SBP-AG, immediately after the procedure, and at discharge. On D2, hemodynamic monitoring was done immediately before bronchoscopy, during BAL, immediately after the procedure, and at discharge. Bars represent median with 25 and 75^th^ percentiles for subjects challenged with RW (white), HDM (gray), CAT (black). Whisker lines represent 10 and 90^th^ percentiles. *p<0.05 compared to value for respective allergen group immediately before procedure. NS indicates there were no significant differences among allergen groups for the indicated time point.

Within each group, there was a modest decrease in SpO_2_ and increase in heart rate during BAL and SBP-AG on D0 and during BAL on D2 (Figure 1). These changes persisted immediately after bronchoscopy but had returned to baseline prior to discharge. At each time point, there were no differences among the three different allergens with respect to changes in either SpO_2_ or heart rate.

### Spirometry

**Figure 2:**
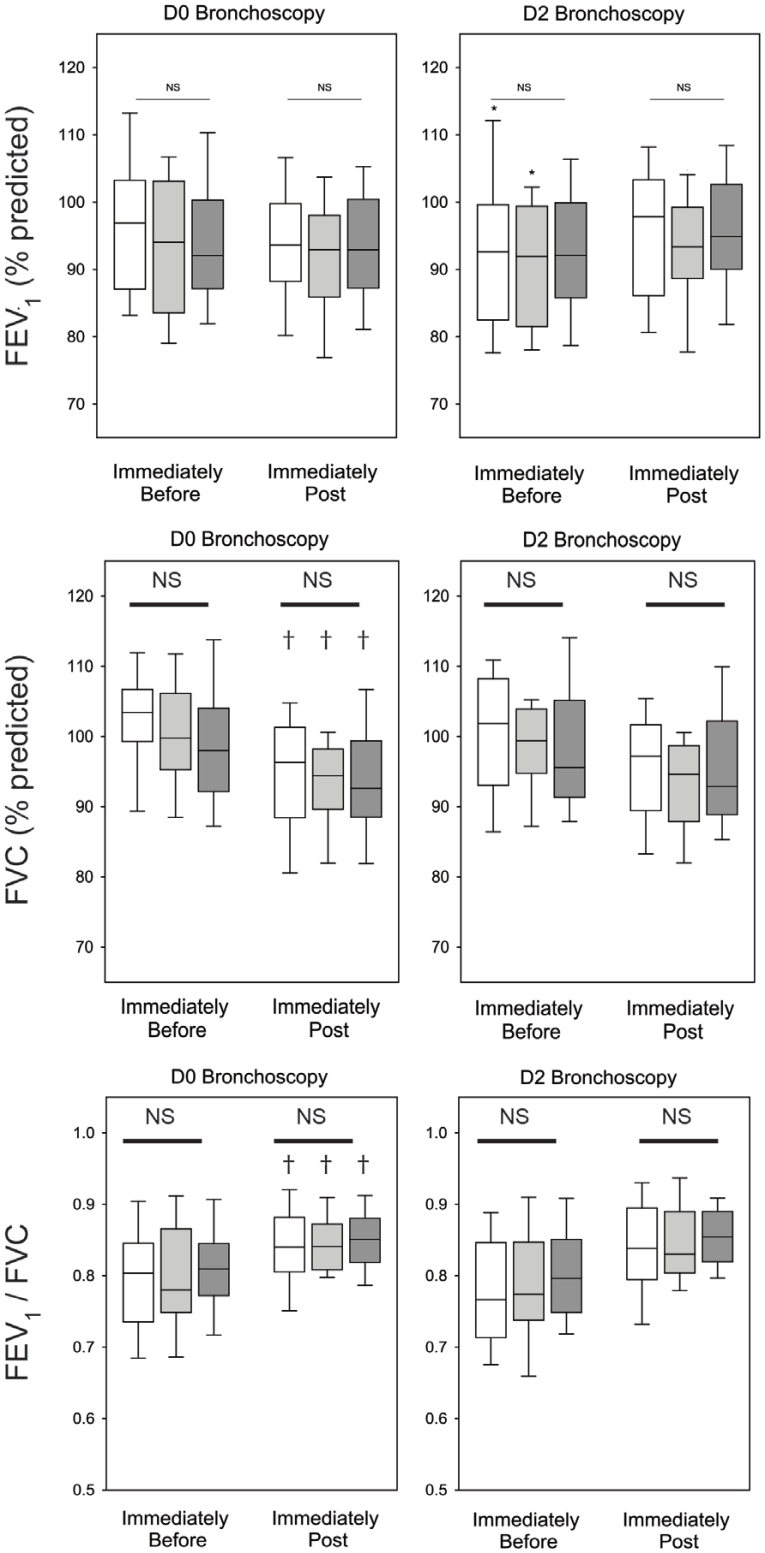
Comparison of FEV_1_, FVC, and FEV_1_/FVC on D0 and 48 hours after SBP-AG (D2) pre-BAL and immediately after BAL. Data are from subjects challenged with RW (white), HDM (gray), or CAT (black) allergen. Bars represent median with 25 and 75^th^ percentiles for challenged subjects. Whisker lines represent 10 and 90^th^ percentiles. *p<0.05 for D2 pre-BAL compared to D0 pre-BAL for respective allergen group, ^†^p<0.05 for D0 post-BAL compared to D0 pre-BAL for respective allergen group. NS indicates there were no significant differences among allergen groups for the indicated time point.

**Figure 3:**
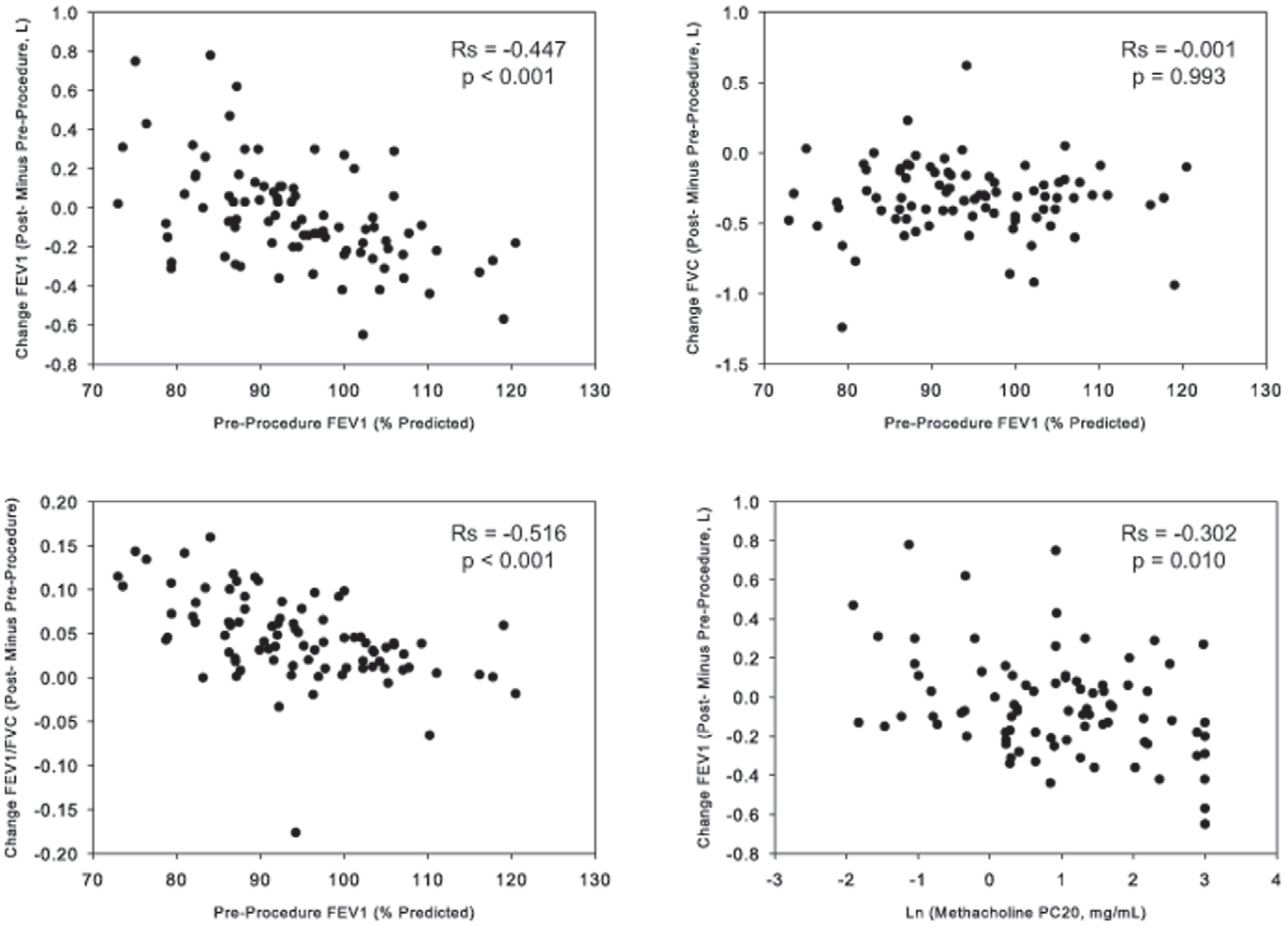
Change in lung function immediately after SBP on D0. The absolute changes in FEV_1_, FVC, and FEV_1_/FVC are plotted for all subjects with negative values reflecting procedure-associated declines. Spearman correlation coefficients are shown, excluding the 10 replicate procedures (n = 77, with the replicate subjects’ earliest datasets included.

None of the allergens was associated with a significant decline in FEV_1_ post-SBP-AG, whereas all three were associated with a decline in FVC and a concomitant increase in FEV_1_/FVC on D0. The changes in FEV_1_, FVC or FEV_1_/FVC were similar among the three allergens (Figure 2). Baseline FEV_1_ on D2 was lower than D0 (p = 0.0005); however, the absolute difference between D2 and D0 was less than 200 mL. Post-procedure FEV_1_ obtained on D2 was similar to that from D0. There were inverse correlations between the change in FEV_1_ (and FEV_1_/FVC) with the baseline FEV_1%_ predicted, such that individuals with greater baseline FEV_1_ had larger declines in FEV_1_ associated with SBP-AG (Figure 3). The decline in FVC was independent of baseline FEV_1_% predicted. A small inverse correlation was observed between the change in FEV_1_ associated with SBP-AG on D0 and the baseline PC_20_ to methacholine (Figure 3).

### Symptoms

Cough was the most common symptom reported immediately after each bronchoscopy. Cough, sore throat, and fatigue were the most frequently reported symptoms at 12 and 24 hours after bronchoscopy. At 24 hours, fever and muscle aches were reported but infrequently (Table 2). There were no statistically significant differences in symptom occurrence or severity among different allergen groups. No subject required prednisone therapy following bronchoscopy.

**Table 2:** Symptoms during and after bronchoscopy.

	During BALRW/HDM/CAT	During SBPRW/HDM/CAT	Immediately PostRW/HDM/CAT	12h PostRW/HDM/CAT	24h PostRW/HDM/CAT
Cough	40/69/63	48/66/63	60/59/67	13/11/17	26/20/8
Chest Tightness	0/0/0	0/3/0	0/6/13	0/4/7	0/3/12
Wheezing	0/0/0	0/0/0	4/3/3	0/15/14	13/7/8
Dyspnea	0/6/0	0/0/0	0/9/0	4/4/3	4/3/0
Chest Pain	0/0/0	0/0/0	4/9/0	0/0/0	0/3/0
Sore Throat	0/0/0	0/0/0	32/19/37	46/63/62	22/13/28
Nasal Stuffiness	0/0/0	0/0/0	0/0/3	4/4/0	4/10/4
Sneezing	0/0/0	0/0/0	4/6/3	NA	NA
Nosebleed	0/0/0	0/0/0	0/9/0	4/0/3	0/0/0
Gagging	4/3/10	0/0/3	NA	NA	NA
Emesis	0/0/7	0/0/0	0/0/0	0/0/0	0/0/0
Fatigue	NA	NA	8/6/7	21/19/14	13/7/0
Headache	NA	NA	4/0/0	17/7/7	0/13/4
Flushing	0/0/0	0/0/3	NA	NA	NA
Dizziness	0/3/0	4/0/0	0/3/0	0/4/0	0/0/0
Fever	NA	NA	NA	0/0/0	9/3/0
Muscle aches	NA	NA	NA	0/0/0	4/0/0

1 Data reflect the percent of subjects for each of the three administered allergens. Symptoms not available for 1 RW, 4 HDM, and 1 CAT procedures 12 h post bronchoscopy; and 2 RW, 2 HDM, and 5 CAT procedures 24 h post bronchoscopy.

(Percentage of subjects reporting each symptom among RW [n = 25], HDM [n = 32] or CAT [n = 30])^1.^

### BAL Fluid Analysis

**Figure 4:**
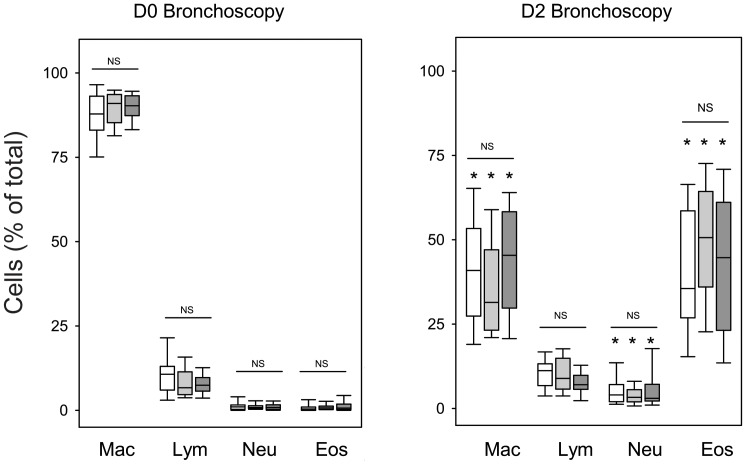
Comparison of BAL on D0 and 48 hours after SBP-AG (D2). Data reflect cells as a percentage of total cells for subjects challenged with RW (white), HDM (gray), or CAT (black) allergen. Bars represent median with 25 and 75^th^ percentiles. Whisker lines represent 10 and 90^th^ percentile. *p<0.05 for D2 compared to D0 for respective allergen group. NS indicates there were no significant differences among allergen groups for the indicated cell type.

Volume recovery of BAL fluid was similar on D0 and D2 and among the different allergen groups. One subject had marginal BAL fluid return (24 ml) on D2 related to poor bronchoscope seal-suction, and was not included in further BAL fluid analysis results. Excluding this subject, the percent recovery of BAL fluid on D0 versus D2 was 73 (69, 76) versus 70 (66, 79) for the RW group, 73 (67, 77) versus 76 (71, 80) for the HDM group, and 73 (69, 76) versus 76 (71, 82) for the CAT group. Total numbers of BAL cells increased 48 hours after SBP-AG, but there were no statistically significant differences among the different allergen groups. Total numbers of BAL cells (x10^4^) per mL of BAL fluid (medians with quartiles) on D0 versus D2 were 13 (10, 16) versus 54 (31, 234) for the RW group, 14 (11, 17) versus 103 (47, 158) for the HDM group, and 12 (8, 13) versus 47 (20, 92) for the CAT group. There was a statistically significant increase in eosinophils and neutrophils as well as a corresponding decrease in percentage of macrophages (Figure 4) without differences in cellular profiles among three allergen groups. The cell concentrations for each population are shown in Table S1.

## Discussion

The ability to directly instill allergens in the lower airway, and evaluate the cellular response thereafter, provides a unique approach to evaluating the pathophysiology of allergic airway inflammation. While the technique of SBP-AG is accepted research tool, there are limited published data regarding its safety, and no publications comparing BAL cellular response or safety profile among different allergens. As with prior reports from other groups [7], [8], [13] there were no major complications associated with bronchoscopy or SBP-AG in our study. However, in our study there were significantly fewer adverse events and a smaller degree of spirometry changes post SBP. There was a modest decline in SpO_2_ and an increase in heart rate during the procedure that returned to normal by the time of discharge. Post-SBP-AG symptoms were mild and transient. Thus, the procedure was well tolerated. The SBP-AG-associated modest decline in FVC and increase in FEV_1_/FVC suggest that the allergen administration led to a restrictive defect probably related to the intense inflammatory response in the challenged segment. Interestingly, the study by Moore et al. evaluating the effects of fixed-dose SBP on subjects with mild-to-moderate asthma showed a greater decline in FEV_1_ and a trend toward reduction in FEV_1_/FVC [7]. The inverse correlation between the change in FEV_1_ and the baseline FEV_1_ percent predicted (Figure 3) was also observed in the bronchoscopy study from the Severe Asthma Research Program evaluating subjects with a wider range of baseline lung function [14]. Nonetheless, our safety data for SBP-AG pertain exclusively to subjects with mild disease not requiring inhaled corticosteroids, and these findings cannot be directly translated to subjects with more severe asthma.

Our study provides a unique addition to the limited existing literature on safety of, and response to, SBP-AG especially comparing different allergens. We evaluated the safety of SBP-AG using a protocol with subject-individualized allergen dosing and compared the safety of bronchoprovocation among three different allergens. There were no differences in either symptoms or objective measures among subjects who received RW, HDM, or CAT allergens. In addition, while there was a marked inflammatory response in BAL fluid on D2 with a predominance of eosinophils, there were no differences in the elicited responses among the three allergens. While there were no differences in the safety profile or cellular responses among subjects challenged with the three allergens utilized in our protocol, it is unclear if these results can be generalized to other allergens that could be used in such procedures.

The low incidence of side effects in our study might be, in part, due to the use of individualized allergen dosing for SBP-AG, which is consistent with the report by Julius and colleagues. In their study, when the allergen dose was individualized based on inhaled allergen testing, only 19% required bronchodilator therapy in the immediate post-challenge period; in contrast, when the allergen dose was not subject-specific, 43% of the subjects required β-agonist treatment. It is important to note that in our study we did not use a fixed dose approach, thus direct extrapolation as to the importance of this approach is not feasible. Compared to other methods used for dose selection such as skin prick titration, the utilization of whole-lung allergen inhalation challenge to determine individual dose for SBP-AG allows for monitoring of the subject’s airway response to allergen challenge with direct relevance to their specific response to SBP-AG. In addition, subjects with highly reactive airways to methacholine were also very reactive to inhaled allergen and as such received a smaller dose of allergen during SBP-AG by this dosing method. Since allergen dose is potentially an important factor in the fall in FEV_1_ after SBP-AG bronchoscopy, this may explain the observed negative correlation between the change in FEV_1_ post bronchoscopy and methacholine PC_20_ (Figure 3). Of note is that subjects who did not tolerate whole-lung allergen inhalation challenge well during screening were not considered for subsequent SBP-AG.

All of our subjects received β-agonists by protocol prior to bronchoscopy and none of the bronchoscopies were discontinued due to wheezing or other acute symptoms during the SBP-AG. While there was a statistically significant reduction in pre-bronchoscopy FEV_1_ on D2 compared to baseline FEV_1_ on D0, the absolute difference between these study visits was less than 200 mL, a change that is not considered clinically significant. The modest reductions in spirometry noted in our study stands in contrast to those reported by Krug et al. [6]; 29% of their subjects developed significant wheezing during the procedure and showed a significant decrease in FEV_1_ following the procedure. Additionally, the drop in lung function was severe enough in half of those subjects to warrant direct instillation of β-agonist bronchoscopically and termination of the procedure. Other studies have also reported larger reductions in FEV_1_ compared to those observed in our study [7], [15], [16]. The reasons that may explain the differences between our findings and those in previously published reports are not clear. Subject selection, recruitment of a relatively younger subject cohort with mild asthma, consistent premedication with β-agonist, use of minimal sedation, differences in total allergen dose, or tailoring allergen dose to each subject based on their individual response to whole-lung allergen inhalation are all among the possible explanations. We should note that in our study, three subjects did not undergo the D2 bronchoscopy. One subject developed sore throat, nasal congestion, and chest tightness approximately 36 hours after the procedure, which improved after β-agonist therapy. The second subject developed fevers, myalgias, and sore throat 24 hours after the procedure that resolved without further intervention and were consistent with acquisition of a naturally occurring viral infection, although these symptoms also could have been related to post-bronchoscopy fever. The third subject developed anxiety accompanied by hyperventilation during the initial procedure, but no changes in objective parameters were seen. This complication was also noted by Julius and colleagues [8]. Finally, there have been safety concerns raised regarding repeated allergen exposures [17]. We did not observe any increase in adverse events in subjects who underwent 2 or 3 SBP-AG studies. This is very consistent with the reports by Julius and Moore [7], [8]. However, this point was not specifically investigated in our study.

In conclusion, our results support the safety of research bronchoscopy with SBP-AG in subjects with mild asthma. Furthermore, we demonstrated similar BAL cellular responses to three different allergens. These data support the continued application of this research tool to the investigation of the mechanisms of allergic airway inflammation in asthma.

## Supporting Information

Table S1 Bronchoalveolar lavage cell concentrations before and after SBP for all antigens combined.
(DOCX)

Text S1 Supplemental Methods.
(DOC)
