# Supplementary material for: Safety of and Cellular Response to Segmental Bronchoprovocation in Allergic Asthma
Source: PLoS One. 2013 Jan 16;8(1):e51963. doi: 10.1371/journal.pone.0051963 (PMC3547018; doi:10.1371/journal.pone.0051963)
Supplement: Table S1 — Bronchoalveolar lavage cell concentrations before and after SBP for all antigens combined. (DOCX) [file pone.0051963.s001.docx]

Table S1. Bronchoalveolar lavage cell concentrations before and after SBP for all antigens combined.

|  | Day 0 (cells*10^3^/mL, median [quartiles]) | Day 2 (cells*10^3^/mL, median [quartiles]) |
| --- | --- | --- |
| Alveolar Macrophages | 112 [88, 131] | 230 [160, 409] |
| Lymphocytes | 9 [5, 16] | 44 [27, 116] |
| Neutrophils | 1 [0.6, 2] | 23 [12, 49] |
| Eosinophils | 0.7 [0.3, 2] | 304 [76, 663] |
