# Supplementary material for: Safety of and Cellular Response to Segmental Bronchoprovocation in Allergic Asthma
Source: PLoS One. 2013 Jan 16;8(1):e51963. doi: 10.1371/journal.pone.0051963 (PMC3547018; doi:10.1371/journal.pone.0051963)
Supplement: Text S1 — Supplemental Methods. (DOC) [file pone.0051963.s002.doc]

**Safety of and cellular response to segmental bronchoprovocation in allergic asthma.**

**Supporting Information (Text S1)**

Loren C. Denlinger, M.D., Ph.D.,^1^ Elizabeth A. B. Kelly, Ph.D.,^1^ Ann M. Dodge, M.S., R.N.,.N.P.,^1^ John G. McCartney, M.D.,^1^ Keith C. Meyer, M.D.,^1^ Richard D. Cornwell, M.D.,^1^ Mary Jo Jackson, R.N., B.S.N.,^1^ Michael D. Evans, M.S.,^2^ and Nizar N. Jarjour, M.D.^1^

From the ^1^Allergy, Pulmonary and Critical Care Division, Department of Medicine and the ^2^Department of Biostatistics and Medical Informatics, University of Wisconsin, Madison, WI 53792

**Supplemental Methods**

**Subject Characterization**

Subjects underwent a thorough health history, physical examination, allergen skin prick test, and pulmonary function testing including spirometry, reversibility to β-agonist, and methacholine challenge. All subjects had a positive skin-prick test to RW, CAT, or HDM, were nonsmokers, and did not have a respiratory infection within 30 days of the study. Subjects were instructed not to use antihistamines for 1-7 days (based on specific preparation), or β-agonists for 6 hours prior to the bronchoscopy. Entry criteria included the absence of corticosteroids or leukotriene antagonists as directed by the primary care provider for 30 days prior to study enrollment.

**Safety Data Collection**

Subjects were closely monitored during and, for at least two hours, following bronchoscopy. Oxygen saturation and heart rate were monitored pre-procedure, continuously during bronchoscopy, and for 30 minutes post-procedure. Supplemental oxygen by nasal prongs was used if the oxygen saturation fell below 90%. The lowest SpO_2_ and highest heart rate recorded during BAL and SBP-AG were used for analysis. Spirometry was performed immediately before and approximately one hour after each bronchoscopy, utilizing American Thoracic Society standards. Discharge criteria included FEV_1_ within 15% of pre-bronchoscopy, pre-albuterol baseline FEV_1_, heart rate < 100, and SpO_2_ > 90% on room air. On discharge, subjects were provided with a peak flow meter, diary card, an albuterol inhaler, and 24-hour emergency contact numbers. Subjects were asked about their symptoms immediately following bronchoscopy; symptoms 12 hours and 24 hours after bronchoscopy were obtained via telephone contact.

**BAL Processing**

BAL was performed with serial aliquots of 40 mL of 0.9% saline solution warmed to 37ºC. A total of 3-4 aliquots were instilled and sequentially aspirated in each segment during each bronchoscopy. BAL samples were processed as previously described [12]. Total BAL cell numbers were determined by hemocytometer using Turk’s counting solution containing acetic acid and methylene blue. For differential cell counts, cytospin preparations of BAL cells were stained with the Wright-Giemsa-based Hema 3 system (Fisher Diagnostics) and are enumerated in Table S1.
